# Feasibility of incorporating the Pocket colposcope into nurse-led cervical cancer screening programs in Western Kenya

**DOI:** 10.3332/ecancer.2025.1925

**Published:** 2025-06-11

**Authors:** Sergine Cindy M Zeufack, Jackton Omoto, Antony Owaya, Everlyn Adoyo, Mercy Rop, Cirillus Ogollah Osongo, Lisa Rahangdale, Craig R Cohen, Chemtai Mungo

**Affiliations:** 1 School of Medicine, University of California, San Francisco, CA 94143, USA; 2 Department of Obstetrics and Gynecology, Maseno University School of Medicine, Kisumu 40100, Kenya; 3 Kenya Medical Research Institute, Kisumu 40100, Kenya; 4 Department of Obstetrics and Gynecology, School of Medicine, University of North Carolina at Chapel Hill, Chapel Hill, NC 27599, USA; 5 UNC Lineberger Comprehensive Cancer Center, Chapel Hill, NC 27599, USA; 6 Department of Obstetrics, Gynecology and Reproductive Sciences, University of California, San Francisco, CA 94158, USA

**Keywords:** cervical cancer, screening, cervicography, digital health, sub-Saharan Africa, acceptability, feasibility

## Abstract

The use of handheld colposcopy in nurse-led cervical cancer screening programs could enhance screening quality and diagnostic accuracy, especially in high-burden regions like sub-Saharan Africa. This study assessed the feasibility of using the Pocket colposcope (Calla Health Foundation, Durham, NC) among healthcare providers in western Kenya. Feasibility was defined in terms of acceptability and image quality. A mixed-methods approach was employed, involving healthcare providers from three clinics offering cervical cancer screening. These providers used the Pocket colposcope more than once within 2 months of the survey. Semi-structured interviews were conducted to explore provider experiences and the device’s acceptability, which was measured using Likert scale questions based on Sekhon’s Theoretical Framework of Acceptability (TFA). This framework evaluates affective attitude, burden, ethicality, perceived effectiveness, intervention coherence, self-efficacy and opportunity cost. Additionally, expert gynecologists assessed the quality of cervical images from visual inspection with acetic acid and/or human papillomavirus-positive patients (*n* = 123), rating them on a scale of 1 to 3. Interobserver agreement was assessed using Cohen’s kappa. Quantitative and qualitative data were analysed using STATA 18.0 and Dedoose 9.0.17, respectively.

Among the eight providers interviewed, including five nurses, the average experience in cervical cancer screening was 4.2 years. The Pocket colposcope received a mean acceptability score of 4.18 (SD = 0.27) out of five based on the seven TFA constructs. Qualitative findings highlighted positive aspects, such as better cervix visualisation, improved post-treatment monitoring, easier external consultations and enhanced patient education. However, challenges incl‑uded camera and software issues and limited personnel for documentation. Image quality was rated 2.23 out of 3, with fair interobserver agreement (Cohen’s kappa 0.36, *p* < 0.001). The study concluded that the Pocket colposcope is acceptable in nurse-led cervical cancer screening programs but emphasised the need for improvements in image quality and technology reliability. Expanding training could further enhance its utilisation.

## Introduction

Globally, cervical cancer remains the fourth most common cancer among women in both incidence and mortality [1]. More than 85% of cervical cancer cases occur in low- and middle-income countries (LMICs) in Africa, Latin America, the Caribbean and Asia [2]. The eastern Africa region has the highest age-standardised incidence and mortality rates, 40.1 and 28.6 per 100,000 women, respectively [3]. In Kenya specifically, cervical cancer contributes to about 13.1% of new cancer cases and is the country’s leading cause of cancer deaths [1]. The Lake Region Economic Block (LREB), 14 counties around Lake Victoria in western Kenya, faces a large burden of cervical cancer morbidity and mortality [4]. Despite existing cervical cancer campaigns and programming, Kenya has low screening coverage and poor linkage to treatment [5]. National studies from 2011 to 2020 reported annual screening coverage between <1% and 36% and precancerous lesion treatment compliance of 22%–39% [5].

The World Health Organisation recommends the ‘screen-and-treat’ approach in LMICs with cervical cancer screening using a human papillomavirus (HPV) test followed by triage with visual inspection with acetic acid (VIA) [6]. In VIA with the naked eye, 3%–5% acetic acid is applied and left on for 1 minute before changes to the cervix are observed without magnification [6]. Based on national screening guidelines, Kenyan providers are counseled to use HPV testing as the primary method for women 30 years and older if available, if HPV testing is not available, VIA is used [7]. VIA or visual inspection with Lugol’s Iodine is the recommended primary test when HPV testing is unavailable or there are concerns of loss to follow up [7]. However, VIA interpretation with the naked eye is highly subjective and dependent on the provider’s level of expertise.

Colposcopy, lower genital tract examination under illumination and magnification [8], is a standard part of cervical cancer screening in high-income countries but has limited availability in LMICs [9]. Multiple studies from low-resource settings have shown that high-resolution digital imaging of the cervix improves the accuracy of VIA-based screening relative to the naked eye [10, 11]. Cervical images, taken by colposcope or a standard camera during screening, can be used effectively for quality control [12] and peer-to-peer learning opportunities [13]. A study in Tanzania found that after Smartphone-enhanced VIA implementation, VIA-positive rates increased from 4% to 6.2%, in a context where VIA-positivity rates for unscreened women are expected to range between 5% and 10% or up to 20% if Human immunodeficiency virus (HIV)-positive [14].

A portable, easy-to-use colposcope presents an opportunity to improve the quality of cervical cancer screening in limited resource settings with benefits such as magnification and a reduced training level required for use [15]. This ability for real time or later feedback strengthens provider capacity for quality control. The ability to connect the portable colposcope to smart devices (i.e., phones and tablets) and save images also allows for remote expert clinician review in real-time or later review for quality control [15]. Compared to standard colposcopes, hand-held colposcopes like the Pocket colposcope have been shown to be an efficient alternative, producing images of medium to high quality [16] with comparable interpretation by expert colposcopists at a fraction of the cost [17].

For the purposes of this paper, nurse-led screening entails nurses as the main screening providers – performing VIA and/or HPV testing, capturing and uploading cervical images to the database and encouraging patients in clinics to undergo cervical cancer screening. Despite the potential benefits of incorporating portable colposcopy in ongoing nurse-led screening programs, more data is needed on the feasibility of their use in LMIC settings, including in western Kenya. Given the need for stakeholder involvement in sustainable interventions, understanding the experiences of staff and their patients is paramount to the sustainable uptake of an intervention such as using the Pocket colposcope. More recently, Sekhon *et al* [18] developed the Theoretical Framework of Acceptability (TFA), which includes the seven constructs of self-efficacy, perceived effectiveness, opportunity costs, intervention coherence, ethicality, burden and affective attitude, as a means of operationalising acceptability in healthcare interventions. The TFA has been applied to healthcare interventions ranging from type 2 diabetes prevention [19] to the extension of cervical cancer screening using HPV testing [20].

This study aims to provide in-depth qualitative insights into provider experience with the Pocket colposcope and assess the quality of the images taken during screening in an LMIC setting. In understanding the acceptability of the tool among providers, we endeavor to evaluate the potential of its more widespread use in the region and similar limited resource contexts. In addition to evaluating acceptability, the assessment of image quality is important to determine the utility of the images for diagnosis and consultation.

## Methods

This study was nested in the LREB study, a 1-year cervical cancer screening project in 2023 of VIA or HPV-positive women in Siaya County. In 2019, Siaya County had a population of approximately 993,183, of which 521,496 were female [21]. Participants screened were women 25 years and older who received services at a participating health facility, Bondo Subcounty Hospital, Siaya County Referral Hospital and Matibabu Foundation Hospital. These three hospitals incorporated the Pocket colposcope into their nurse-led cervical cancer screening program. The providers received a few hours of virtual and/or in-person training to learn theory and practice Pocket colposcope use.

**Figure 1.**
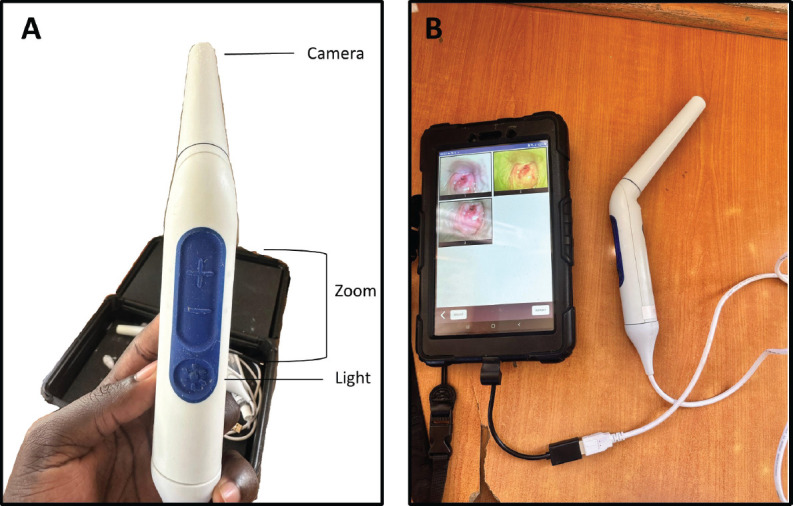
Pocket colposcope. (a): Annotated image of the Pocket colposcope. (b): Pocket colposcope attached to a tablet with cervical images using both white and green light.

The Pocket colposcope possesses a camera, magnification features and white and green lights for optimised image capture during screening (Figure 1). The green light filter allows for better visualisation of cervical vasculature [15]. During screening, providers collected images using the Pocket colposcope. The Pocket colposcope is designed to be inserted through a speculum [16]. After the speculum was inserted, 10% acetic acid was applied to visualise lesions and the Pocket colposcope was inserted into the vaginal canal to capture 2–8 cervical images 1 minute after application. The Pocket colposcope was sterilised between patients. For participants with eligible precancerous cervical lesions, images were taken before and after on-site thermal ablation in line with ‘screen-and-treat’ guidelines [6].

### Image quality assessment

If more than three images were available for a participant, the best three were selected for image quality assessment. Images were arranged and ranked in columns. The first two columns were images captured with standard white light. The third column was a green light image, if available, or another white light photo, if not.

Image ranking was completed by two expert Obstetrics and Gynecology physicians with significant (more than 10 years) experience in colposcopy and cervical image review at accredited health facilities. The scoring system was developed using criteria from studies in similar limited-resource contexts [16, 22, 23]*.* Images were scored and ranked as low quality (score = 1), medium quality (score = 2) and high quality (score = 3). Low quality was defined as images that were not in focus and/or lack visibility of most (3 out of 4) cervical quadrants. Medium quality was defined as images slightly out of focus but with most quadrants visible. Finally, high-quality images will be those where the image was in focus and all quadrants are visible. The images were organised into an image bank based on scoring independently assigned by each observer.

### Healthcare provider acceptability

An acceptability questionnaire was developed by the primary author and pilot tested by one of the non-clinical members of the research team who had used the Pocket colposcope. The questionnaire was subsequently modified based on feedback. To assess acceptability, all healthcare providers at the three participating health facilities who had used the Pocket colposcope within 2 months and at least twice were eligible for the interview. Eight healthcare providers met these criteria and were approached by telephone to meet for a one-on-one in-person interview to answer the acceptability questionnaire. Interviews were conducted in English in July 2023 at the health providers’ respective facilities. Seven providers consented to an audio recording of the interview for transcription.

The same interview guide was used for each interview. The first part of the survey included questions on sociodemographic data and open-ended questions on training, benefits and disadvantages of the Pocket colposcope, recommendations and patient reactions. The primary author, who is a current medical trainee, was the interviewer; the interviewer shared racial but not ethnic concordance with the providers. The providers had not met the interviewer prior to the interview. Qualitative findings were shared with providers after qualitative analysis was completed.

**Figure 2.**
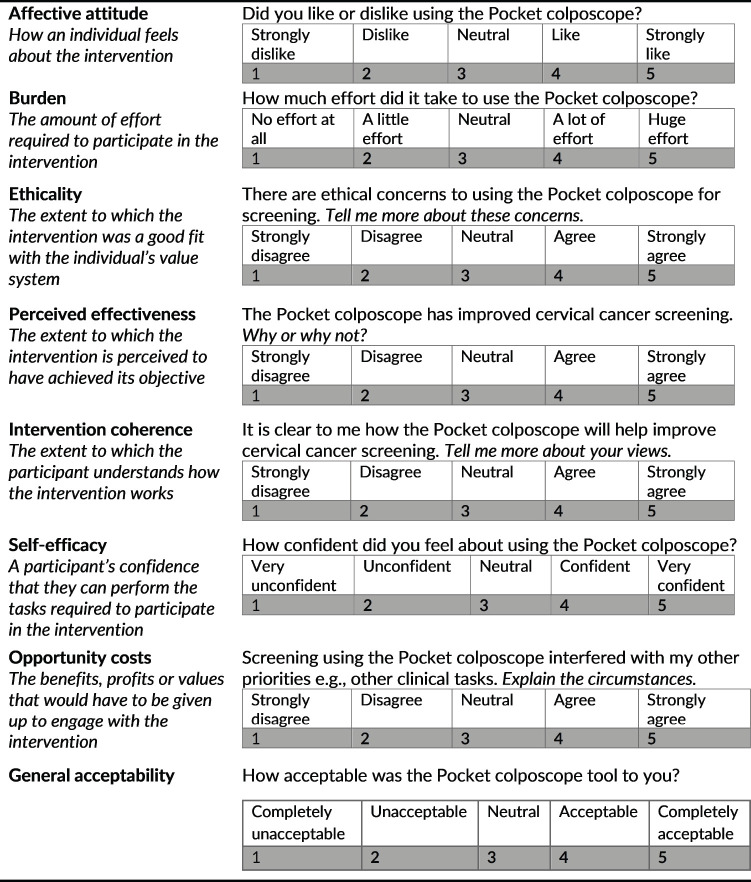
Questions based on Sekhon’s TFA. Constructs modified from Sekhon’s TFA and their associated Likert-scale questions.

The second part of the survey included Likert-scale questions based on Sekhon’s seven TFA constructs [18]: affective attitude, burden, ethicality, perceived effectiveness, intervention coherence, self-efficacy and opportunity costs (Figure 2). The providers were also provided with a general acceptability question. Per Sekhon’s framework, general acceptability was calculated using the general acceptability score and the average of the seven constructs. The Likert-scale questions were supplemented with open-ended questions to provide nuance and all responses were recorded and manually transcribed verbatim. When calculating the scores from the TFA constructs, the items for burden, ethicality and opportunity costs were reverse scored so that a higher score indicated increased acceptability in line with the other constructs.

### Data analysis

Quantitative survey data were analysed using STATA version 18.0 and MS Excel. For image quality, the rankings for the two raters were compared using Cohen’s kappa statistic [24] to determine the degree of inter-rater agreement on image ranking. Correlation between the TFA constructs was assessed using Pearson’s statistic. For the qualitative data from the in-depth interviews, inductive content analysis was performed using Dedoose Version 9.0.17 (2021). Two coders independently reviewed and coded the transcripts before collaborating to analyse the main themes.

### Ethical approval

The overall study was reviewed and received ethical approval from the University of North Carolina Chapel Hill (U.S.A.) and Maseno University in Kenya. Written informed consent was obtained from all study participants before participation.

## Results

### Quantitative findings

#### Acceptability survey

The surveys were conducted with eight cancer screening health providers using the Pocket colposcope across all three study sites. All providers were female, and half of the providers surveyed worked at Matibabu Foundation Hospital (Table 1). Most health providers (62.5%) were nurses with a diploma. The average age was about 32 years.

**Figure 3.**
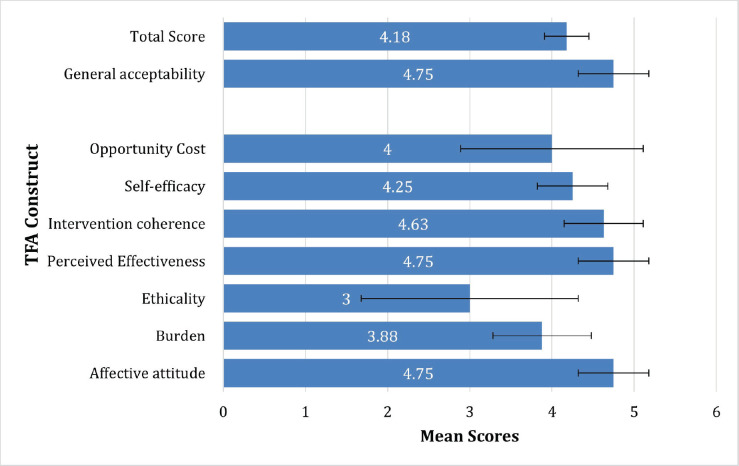
Mean scores of TFA constructs. Mean scores of TFA constructs with standard deviation.

The mean acceptability score using the seven constructs was 4.18 (SD = 0.27) out of 5, and the general acceptability score was 4.75 (SD = 0.43) out of 5 (Figure 3). All constructs other than ethicality had high (>4) average scores. Of the seven constructs, only ethicality and opportunity cost had a variance of >1*.* For the correlation matrix produced with Pearson’s correlation coefficients – the only statistically significant correlation was between the ‘perceived effectiveness’ and ‘intervention coherence’ constructs, *r* = 0.74 (*p* = 0.03). There were no significant correlations observed between scores for the individual TFA constructs and the general acceptability item.

#### Image quality review

**Figure 4.**
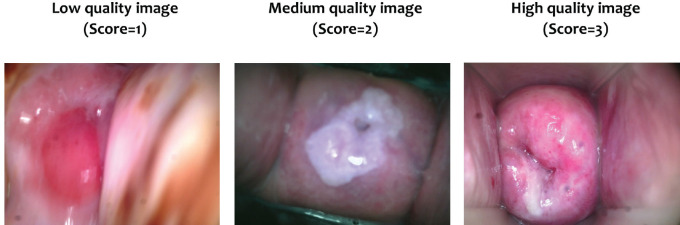
Examples of rated cervical images. Cervical images with agreement with both raters on the assigned score.

Due to troubleshooting and device issues, 117 of the 123 participants (95.1%) had images. A total of 350 images were rated. The average quality rating was approximately 2.23 out of 3, with an average of about 15% of images as low quality (score = 1), 47% as medium quality (score = 2) and 38% as high quality (score = 3) (Table 2). An example of a low, medium and high-quality image, with consistent ratings for both raters, is included in Figure 4.

Interobserver agreement between the raters was assessed with Cohen’s kappa and ranged between 0.33 and 0.36 for the three image columns (Table 3). The calculated Cohen’s kappa for the overall sample (*N* = 350) was 0.36 (SE = 0.04, *p* < 0.001), indicating fair agreement [21].

### Qualitative findings

The transcripts of the seven providers that consented to recording analysed using inductive content analysis and produced seven themes – benefits of the Pocket colposcope, challenges to its use, presence or absence of conflicting priorities, ethical concerns, patient reactions to the tool, acceptability of the tool to other health providers and recommendations for improvement of the Pocket colposcope. The representative quotes are presented by theme and anonymised respondents are denoted with the respondent number (Rx).

### Theme 1: Benefits to the Pocket colposcope

Respondents mentioned five common main benefits of the Pocket colposcope.

1) The ability for external consultation on images – *‘But if you use the colposcope and take an image, then you can have another person have an additional say to what you have seen. Other people can consult, rather than just relying on your own understanding.’ -R6*

2) The use of the images for diagnosis – *‘If it is a VIA positive or it is a suspicious one, the images will be clearly visible.’ -R5*

3) The improved visualisation of the cervix – *‘The advantage of it is that I’m able to visualize the cervix. Initially, you would just use your eyes, but with this Pocket colposcope, you can literally see it from the phone. Being that it’s a camera or something that’s taking a picture, you are able to zoom it, and you can detect even the slightest details compared to the previous visual.’ -R7*

4) The ability to collect and store data for review – *‘You can use it to visualize and collect the data. You can use it to extract the data later of the client.’ -R2*

5) Making treatment decisions based on progression or presence of lesions – *‘That’s why I’m saying it’s very interesting to have such a machine. Because it promotes early treatment and also helps us to monitor the treatment progress of our patients.’ -R4*

For all but 1 of the providers, the benefits of colposcopy were relative to the naked eye. However, one respondent was able to compare the Pocket colposcope to another specialised colposcopy tool, the EVA System [25]. R2 found the Pocket colposcope was better in all aspects, including ease of use and image quality – describing the images as ‘brighter and clearer.’ R2 also mentioned portability and time – finding that ‘the package of the Pocket colposcope [was] easier to transport’ and that screening with the Pocket colposcope took ‘a shorter time.’

### Theme 2: Barriers/Challenges to the Pocket colposcope

The interviews highlighted three critical issues.

1) The hardware, i.e., camera malfunctioning, blurry images and spots on the lens.

*‘Unless the camera fails, at times it fails, you’re already with a client there and then it fails, or it brings a problem.’ -R2*

*‘The one we sent to Kisumu [for repair] sometimes would give us blurry images’ -R5*

2) The software, i.e., app failures and data loss

*‘You click it, and it will just be loading so you have to restart the phone for the app to work smoothly. It takes time. Sometimes you’ll be restarting, and the nurse tells you they have to add another acetic, the lesion has disappeared. So, it interferes with the timing.’ -R5*

3) Logistical challenges, i.e., needing assistance to use the Pocket colposcope while entering data, or the difficulty of sharing only one colposcope for one facility

*‘You cannot operate alone, it will need an assistant so that the assistant can help you during the visualization, enrollment of the client, and during the sample taking. That one is the only disadvantage’ -R4*

*‘If you use the colposcope when you are alone it’s a bit hectic because you have to navigate through a lot of things. But if you have someone there, then it’s a bit easy and fast to use. You cannot do all that when you are alone, you need to have someone to help you, to do all that. So that was the main barrier.’ -R6*

### Theme 3: Opportunity cost

The opportunity cost was defined in this study as interference with other priorities or tasks, which included other clinical tasks for nurses. Those who disagreed that there were conflicting priorities found that it was their ‘priority’ and ‘responsibility’ as providers to care for the patient – regardless of the added task. *‘For me it didn’t interfere with any of my tasks because I was assigned in that line. So, it didn’t interfere with me.’ -R5*

Those who were neutral or agreed found that the added time and the need for additional assistance in the context of staff (namely, nurse) shortage contributed to the cost. *‘At times, we have a shortage of staff so you find, I cannot spend much time, or there’s nobody to assist me. So, at times it interferes.’ -R2.*

### Theme 4: Ethical concerns

The primary concern voiced by those who agreed that there were ethical concerns with the Pocket colposcope was privacy and confidentiality surrounding the imaging. *‘The only ethical concern was that since we are taking the images, are patients aware why we are taking images. Because some are like ‘where are you going to post my images?’ So, we explain to them that this is purely for research and treatment. So that we will not write your name, we will not say this lesion is for so and so and advertise it.’ -R6*

The respondents who did not believe there were ethical concerns thought that potential ethical concerns were adequately addressed by the consent process completed before Pocket colposcope use. *‘I didn’t have any ethical concerns; I explained to the clients- they gave me a go ahead, and there were no ethical concerns.’ -R5*

### Theme 5: Patient reactions from the health provider perspective

All seven providers said their patients mostly reacted positively to the Pocket colposcope. Some patients appreciated the ability to view their cervical images before and after treatment. One respondent voiced how having patient images to visually explain the need for treatment allowed for ‘better understanding’ compared to verbal explanations of cervical lesions. *‘So currently, most of them are really positive about it because they know that they are going to get that help. Sometimes, some request to be shown the images, and when we do that, we are able to explain to them that there is a problem here and everything, compared to when I only explain it verbally and they don’t see it. So, there is a better understanding, so they really appreciate it.’ -R7*

Negative reactions to the Pocket colposcope included apprehension about potential discomfort or pain from the overall colposcopy process. *‘According to the patients that I have attended so far, they don’t have negative experience with the colposcope machine. They only have negative experiences during the biopsy because they will say that it is painful.’ -R4*

Regarding managing patient’s potential negative emotions, respondents reported that counseling before using the Pocket colposcope – ensuring that the patients knew the steps of the colposcopy procedure-helped patients. *‘Before we used the colposcope, we used to explain to them what we were going to do. So, they were okay with it, as long as it was not causing extra pain, they were okay with it.’ -R6*

### Theme 6: Increasing acceptability for other healthcare workers

Per the respondents, the acceptability of the tool for other healthcare workers could be impacted by factors varying from staffing, passion/interest, time and training. One respondent mentioned that among other staff, there was ‘interest in the colposcope and how best they can use it’ (R6), while another cited that other staff would be interested in the tool as a means of supporting their client (R1). *‘For the health providers I think their main goal is just to help the client. And helping the client means getting the exact results, the accurate results, and that means in visualizing the cervix you just have to be sure of what you’re seeing. So, I think they’d accept it, and it would be very useful for them.’ -R1*

Techniques suggested for increasing acceptability for other healthcare workers in the region included increased hands-on training and education about the benefits of the Pocket colposcope. *‘When something is new to you, you’re always not sure. Some people don’t just accept it like that…But I believe with further explanation and training them on how to use it and everything, I feel some of them will loosen up their hearts and try it out.’ -R7*

### Theme 7: Recommendations for improving the Pocket colposcope

The providers gave three main recommendations.

1) Changes to the colposcope – namely the software – *‘The machine itself is good’ what should be worked on is the app.’ -R5*

Though proposed changes to the colposcope were primarily related to the software ‘app,’ one respondent asked for camera improvements for clearer visualisation.

2) Increasing the number of available colposcopes for use – *‘I would recommend even more colposcope machines to be brought in different facilities so that it can help health workers to visualize very well the lesions and also help them to do any other investigation related to the cervix.’ -R4*

3) Increasing the number of trained personnel for logistical improvement – *‘They should train more and more people on usage of it…It should be a longer period of training and then they should train more people.’ -R2*

One respondent mentioned focusing on training staff members in relevant departments such as Maternal and Child Health (MCH) and HIV clinics. Another respondent noted that providing the Pocket colposcope in more local screening clinics could prevent the ‘little lesions’ from progressing to a larger size before patients reach screening providers at the county hospital level.

## Discussion

The study demonstrates the feasibility of using the Pocket colposcope for cervical cancer screening in Western Kenya. Quantitatively, the Pocket colposcope cervical image scores indicated predominantly medium image quality. The Pocket colposcope had high acceptability scores among the eight providers. The image review by the external raters produced a Cohen’s kappa with a fair level of interobserver agreement [26], identical to Fleiss’ kappa calculated for off-site raters using Pocket colposcope images in Peru [16]. Though consistent with prior studies, this kappa indicates potential for improvement, either in terms of clarifying image quality criteria or accounting for the subjectivity of image ratings. The seven interview transcripts detailed five main benefits of the Pocket colposcope – consultation, treatment and monitoring, diagnosis, data collection and improved visualisation. These benefits contrast with three main challenges – hardware and software (camera and app), supply and logistical (limited personnel).

The Pocket colposcope’s ability to allow users to visualise the cervix better and store post-VIA images for future review can strengthen the accuracy of screening and referrals in this setting. From the scores from Sekhon’s TFA, the positive statistically significant correlation between intervention coherence and perceived effectiveness for Pocket colposcope use indicates that provider understanding of how the Pocket colposcope worked correlated with their belief that it achieved its objective of improved screening. The Pocket colposcope could enhance the efficiency and accuracy of medical practices by simplifying the visualisation of cervical images and facilitating diagnosis and invaluable interprofessional consultation.

Given the challenges faced by providers who used the Pocket colposcope, there remains room for improvement to enhance overall user satisfaction and data quality. One main suggestion was to improve the hardware and software; this could include software updates to reduce long loading times or a dedicated team member to assist with troubleshooting. Second, many respondents described using the Pocket colposcope as a two-person job—increasing the number of trained staff could increase the reach and use of the tool and improve workflow. Training staff in MCH and HIV clinics could improve screening rates in target populations. Existing staff could benefit from improved training to ensure effective tool utilisation and improve image quality through better positioning and focus. Chronic staff shortages, however, remain a valid structural barrier to uptake and improvement.

Overall, patient reactions underscore the importance of provider-to-patient communication and addressing patient concerns to ensure a positive experience with the Pocket colposcope. Similarly to a recent Ugandan study on smartphone-based telemedicine use in cervical cancer screening, the explanation and sharing of cervical images allowed for increased patient involvement in the screening process [27]. Thorough provider explanations also addressed concerns about privacy and confidentiality. Though the providers commented on patient reactions, a direct survey of patient attitudes and beliefs would provide another perspective of the Pocket colposcope acceptable from a valuable stakeholder in screening.

The study’s strengths include using a validated framework [18] to assess acceptability and a large sample of photos for image quality assessment. The in-depth interviews supplied better insight into providers’ opinions on the advantages and disadvantages of using the Pocket colposcope. These insights benefit the research team, composed of non-clinical and clinical personnel and provide areas for growth. Given that the providers were primarily nurses, this represents an opportunity to continue task shifting to reduce physician workload while increasing access to screening services. This study is the first in-depth evaluation of Pocket colposcope acceptability among providers involved in cervical cancer screening in Africa. It provides a unique perspective while adding to existing literature on the acceptability and feasibility of digital cervicography in low-resource context screening programs [28]. Notably, the study had no concerns about a lack of electricity as the Pocket colposcope charged when connected to smartphones or tablets, demonstrating the importance of colposcopy tools that do not rely on traditional power sources.

The limitations of our study include a small sample size for the measure of acceptability. There may also be limited depth of understanding of the tool, as the providers had only used the Pocket colposcope for approximately 5 months at the time of the interviews. As such, further benefits or challenges may still need to be elucidated. Additionally, Likert scale self-report questions, such as those from Sekhon’s TFA, have been associated with an increased risk of acquiescence bias and social desirability bias [29]. The assurance of anonymity during and after interviews was used to mitigate acquiescence bias.

## Conclusion

The study shows that the Pocket colposcope is acceptable among primarily nurse health providers in Siaya County, Kenya, with benefits for cervical visualisation and external consultation. The study also highlights barriers to effective use, including the need for improved training and software. If these barriers are addressed, the tool could prove promising for increased use in similar contexts to improve cervical cancer screening efforts. Further studies are indicated to ascertain the feasibility of the Pocket colposcope on a larger scale in the region.

## Conflicts of interest

The authors have no conflicts of interest.

## Author contributions

SCMZ and CM were involved with the conception and design of the research with contributions from JO, AO and COO. SCMZ wrote the questionnaire and conducted the interviews. CRC and LR evaluated and scored the images. SCMZ and EA carried out the qualitative analysis. SCMZ and MR completed the quantitative analysis. SCMZ and CM wrote the manuscript. SCMZ created the figures and tables. CM, LR and CRC edited this manuscript. All authors reviewed the manuscript.

## Figures and Tables

**Table 1.** General characteristics of providers.

Provider characteristics	*Providers (N = 8)*
Gender, n(%)	
Female	8 (100)
Profession, n(%)	
Nurse	5 (62.5)
Physician	1 (12.5)
Research assistant	2 (25.0)
Site, n(%)	
Bondo	2 (25.0%)
Matibabu	4
Siaya	2
Highest level of education, n(%)	
Diploma	5
Bachelors	3
Age, years, mean (±SD)	31.8 (7.0)
Time as clinical provider, years, median (± IQR)	4.5 (3.8)
Time as cervical cancer screening provider, years, mean (±SD)	4.2 ± 4.7

**Table 2.** Average ratings for images.

Observer	Average rating	Low quality *n* (%)	Medium quality *n* (%)	High quality *n* (%)
Rater 1	2.33	52 (14.86)	129 (36.86)	169 (48.29)
Rater 2	2.13	53 (15.14)	200 (57.14)	97 (27.71)
Overall avg	2.23	52.5 (15)	164.5 (47)	133 (38)

**Table 3.** Agreement on cervical image quality.

Category	Kappa	Standard error	*p* value
Image 1 (*n* = 117)	0.33	0.06	<0.001
Image 2 (*n* = 117)	0.37	0.07	<0.001
Image 3 (*n* = 116)	0.36	0.07	<0.001
Overall (*N* = 350)	0.36	0.04	<0.001
